# Supplementary figures and images for: Amorphous Silicon Thin-Film Solar Cells on Fabrics as Large-Scale Detectors for Textile Personal Protective Equipment in Active Laser Safety
Source: Materials (Basel). 2023 Jul 5;16(13):4841. doi: 10.3390/ma16134841 (PMC10343704; doi:10.3390/ma16134841)

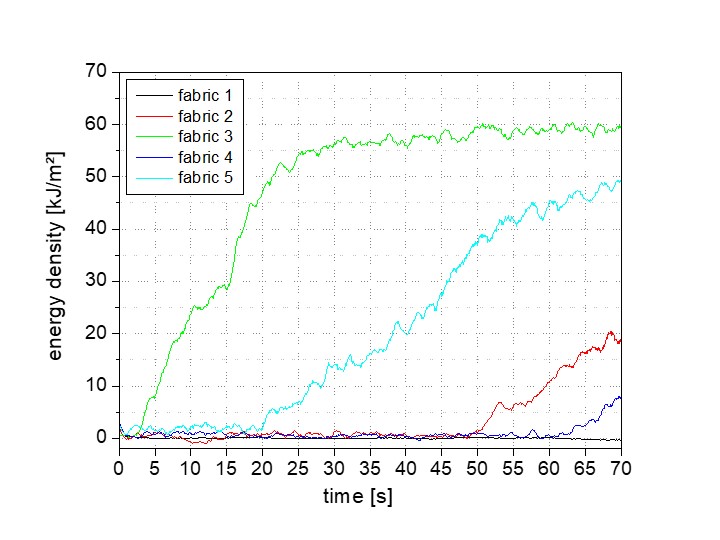

Supplement: Supplementary file 1 [file materials-16-04841-s001.zip › Supplementary files/Figure S1.jpg]

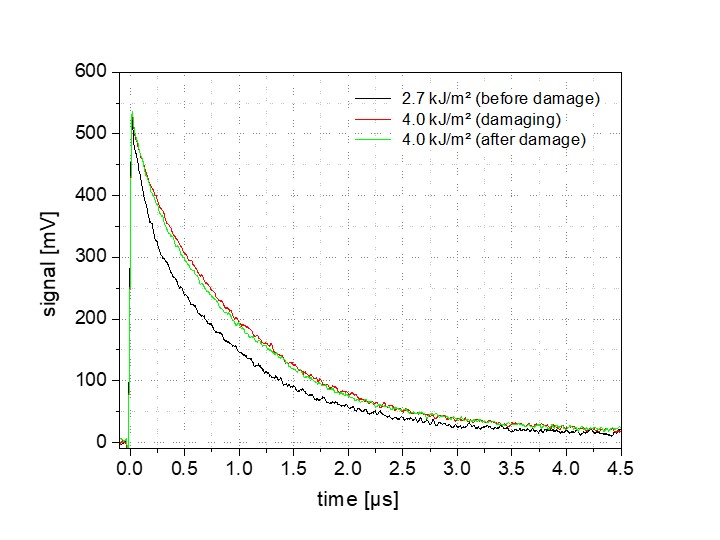

Supplement: Supplementary file 1 [file materials-16-04841-s001.zip › Supplementary files/Figure S2.jpg]

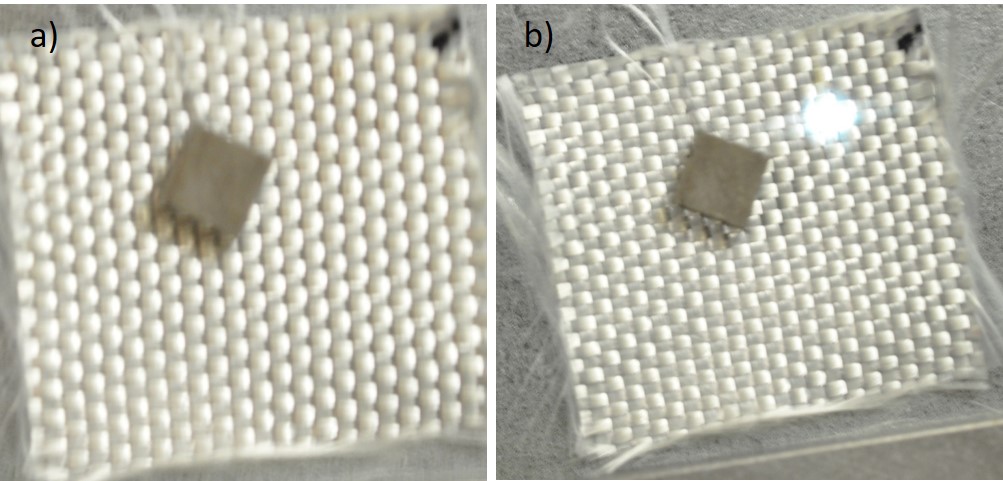

Supplement: Supplementary file 1 [file materials-16-04841-s001.zip › Supplementary files/Figure S3.jpg]

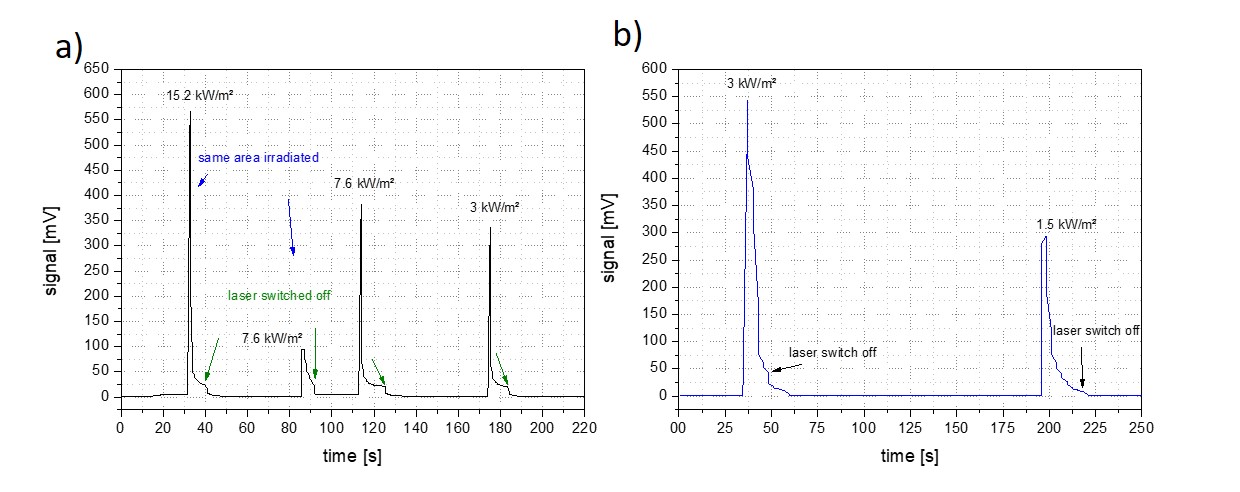

Supplement: Supplementary file 1 [file materials-16-04841-s001.zip › Supplementary files/Figure S4.jpg]
